# Articular chondrocyte-derived extracellular vesicles promote cartilage differentiation of human umbilical cord mesenchymal stem cells by activation of autophagy

**DOI:** 10.1186/s12951-020-00708-0

**Published:** 2020-11-09

**Authors:** Ke Ma, Bo Zhu, Zetao Wang, Peian Cai, Mingwei He, Danyan Ye, Guohua Yan, Li Zheng, Lujun Yang, Jinmin Zhao

**Affiliations:** 1 Guangxi Engineering Center in Biomedical Materials for Tissue and Organ Regeneration, The First Affiliated Hospital of Guangxi Medical University, Nanning, China; 2 Department of Plastic & Cosmetic Surgery, The First Affiliated Hospital of Guangxi Medical University, Nanning, China; 3 Department of Orthopaedics Trauma and Hand Surgery, The First Affiliated Hospital of Guangxi Medical University, Nanning, China; 4 Department of Burns and Plastic Surgery, The Second Affiliated Hospital of Shantou University Medical College, Shantou, China; 5 Research Centre for Translational Medicine, Shantou University Medical College, Shantou, China; 6 Pharmaceutical College, Guangxi Medical University, Nanning, China; 7 International Joint Laboratory of Ministry of Education for Regeneration of Bone and Soft Tissues, The First Affiliated Hospital of Guangxi Medical University, Nanning, China; 8 Guangxi Key Laboratory of Regenerative Medicine, The First Affiliated Hospital of Guangxi Medical University, Nanning, China

**Keywords:** Autophagy, Chondrocytes, Chondrogenesis, Extracellular vesicles, Human umbilical cord mesenchymal stem cells

## Abstract

**Background:**

Umbilical cord mesenchymal stem cell (HUCMSC)-based therapies were previously utilised for cartilage regeneration because of the chondrogenic potential of MSCs. However, chondrogenic differentiation of HUCMSCs is limited by the administration of growth factors like TGF-β that may cause cartilage hypertrophy. It has been reported that extracellular vesicles (EVs) could modulate the phenotypic expression of stem cells. However, the role of human chondrogenic-derived EVs (C-EVs) in chondrogenic differentiation of HUCMSCs has not been reported.

**Results:**

We successfully isolated C-EVs from human multi-finger cartilage and found that C-EVs efficiently promoted the proliferation and chondrogenic differentiation of HUCMSCs, evidenced by highly expressed aggrecan (ACAN), COL2A, and SOX-9. Moreover, the expression of the fibrotic marker COL1A and hypertrophic marker COL10 was significantly lower than that induced by TGF-β. In vivo, C-EVs induced HUCMSCs accelerated the repair of the rabbit model of knee cartilage defect. Furthermore, C-EVs led to an increase in autophagosomes during the process of chondrogenic differentiation, indicating that C-EVs promote cartilage regeneration through the activation of autophagy.

**Conclusions:**

C-EVs play an essential role in fostering chondrogenic differentiation and proliferation of HUCMSCs, which may be beneficial for articular cartilage repair.
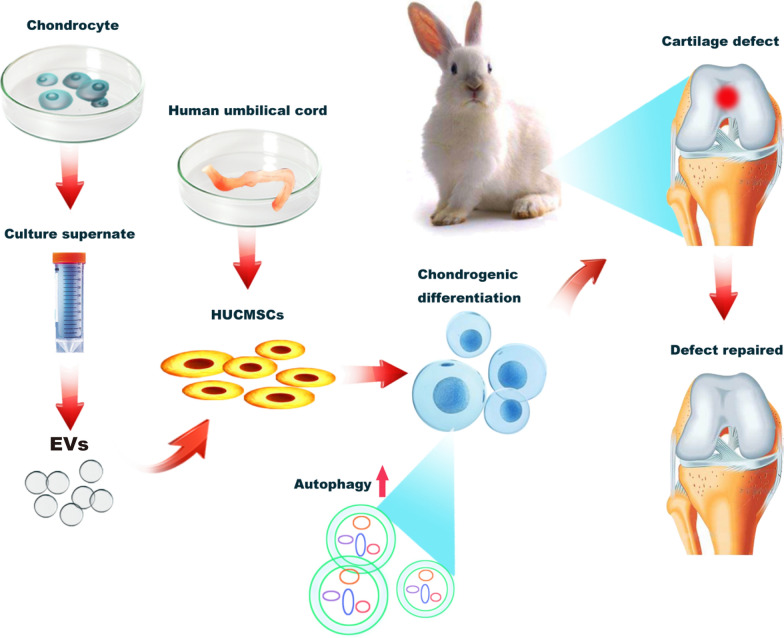

## Introduction

Regeneration of damaged articular cartilage remains a challenge for cartilage repair. In the clinic, current treatment strategies for cartilage injuries, including microfracture and autogenous cartilage transplantation, are not satisfactory. In recent years, stem cell-based strategies using multipotent mesenchymal stem cells (MSCs), which have the ability of self-renewal and can be specifically differentiated into chondrocytes [1, 2], has emerged as a promising modality for cartilage regeneration.

The chondrogenic differentiation of MSCs is profoundly affected by the extracellular microenvironment and various growth factors [3]. TGF-β (Transforming growth factor-beta) is recognised as the main transforming growth factor [4, 5] that has the potential to induce the direct differentiation of MSCs to chondrocytes [6, 7]. However, TGF-β also causes cartilage hypertrophy during the process of cartilage differentiation [5], and the application of TGF-β leads to complications such as functional heterogeneity, degradation, and loss of activity, which ultimately limits its clinical applications. Hence, it is imperative to find alternative small molecular actors that are functionally homogenous for cartilage regeneration.

EVs are a class of nano-scaled vesicles that have been identified as the crucial intermediate cell mediator of intercellular communication through the transfer of bioactive components, including various proteins and microRNAs, thereby affecting the phenotype and function of recipient cells [8–11]. Current studies have found that EVs are released by multivesicular bodies (MVBs) from parental cells [12]. Therefore, EVs reflect the composition of parental cells to a certain extent. According to its source and parental cell status, EVs display a variety of functions such as tissue regeneration, immune regulation, and gene regulation [13–15]. In recent years, the role of EVs in tumours and immunology has been extensively studied. However, the role of EVs as an inducer of stem cell differentiation has rarely been studied. An in vitro study showed that exosomes derived from osteoblasts could promote stem cell differentiation into osteoblasts, while exosomes derived from adipocytes induced adipogenic differentiation of stem cells in an osteogenic medium environment [16]. Since exosomes are a type of EVs, these findings indicate that EVs retain the multiple biological activities of homologous cells [17]. Additionally, rabbit chondrocytes-derived EVs could foster ectopic cartilage formation of cartilage progenitor cells in the subcutaneous environment [18], suggesting that chondrocyte-derived EVs play a crucial role in chondrogenesis. Thus, human articular chondrocytes-derived EVs may be promising substitutes for TGF-β to guide chondrogenic differentiation of MSCs. However, this strategy has not been studied yet.

Among various types of MSCs, human umbilical cord mesenchymal stem cells (HUCMSCs), which are derived from Wharton’s jelly, the primitive connective tissue surrounding umbilical cord vessels, are attracting because they exhibit unique properties of higher proliferation rates, immunosuppression, and lower immunogenicity, as well as relatively easy and non-invasive isolation procedures compared to other sources of derived MSCs [19–21]. Therefore, HUCMSCs are the ideal and noncontroversial cell source for regenerative medicine and hold promise in cartilage regeneration and repair.

In this study, we demonstrated that human C-EVs could promote the proliferation and chondrogenic differentiation of HUCMSCs, which are beneficial to cartilage regeneration in vivo. Chondrogenic differentiation by C-EVs may be associated with the activation of autophagy in HUCMSCs. This study may provide a reference for the clinical application of EVs in cartilage repair.

## Results

### Characterisation of HUCMSCs

**Fig. 1:**
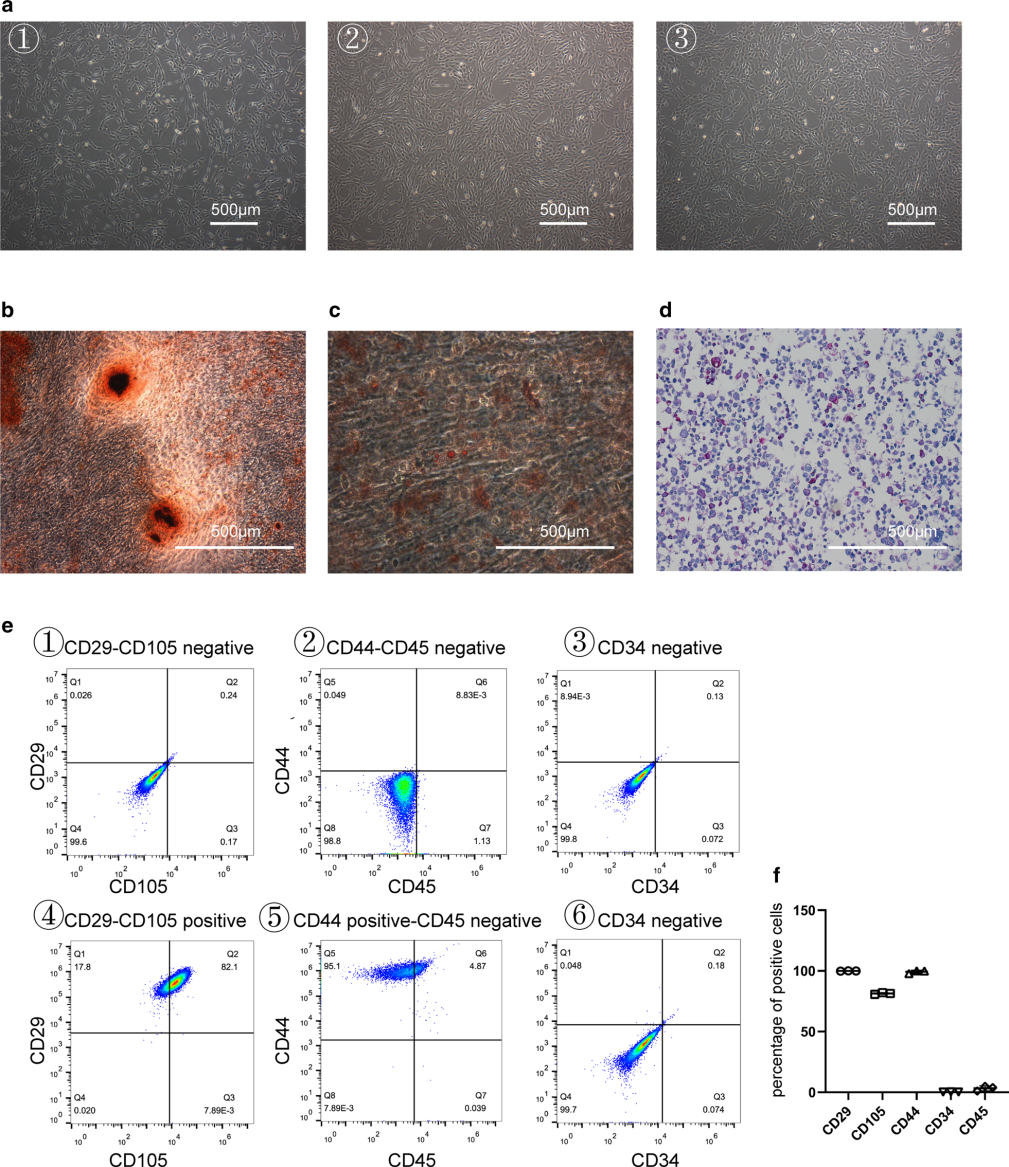
Detection of HUCMSC characteristics. **a** The representative spindle-like morphology of HUCMSCs in passages 3–5 were observed by microscopy. The multipotency of HUCMSCs for osteogenesis, adipogenesis, and chondrogenesis was determined by alizarin red staining (**b**), oil-red-o staining (**c**), and haematoxylin–eosin and alcian blue dual-staining (**d**), respectively. Scale bar = 500 μm. **e** Flow cytometric analysis of mesenchymal-related markers: CD29, CD105, CD44, CD45, CD34. ①②③ were cells without antibodies as negative controls, and ④⑤⑥ were flow cytometry results after adding a fluorescent antibody. This experiment was independently repeated three times. **f** The rates of each marker-positive cells (n = 3)

To examine the morphology of HUCMSCs isolated from human umbilical cord tissue, HUCMSCs at passage 3–5 were observed using optical microscopy. As shown in Fig. 1a, HUCMSCs exhibited a fibroblastic and spindle-shaped morphology, which are typical morphological characteristics of MSCs. Moreover, the multipotency of isolated HUCMSCs was also evaluated by different inductive conditions. The osteogenic, adipogenic, and chondrogenic differentiation of HUCMSCs was verified using alizarin red staining, oil-Red-O staining, and alcian blue staining, respectively. A high degree of mineralisation, intracytoplasmic lipid droplet accumulation, and positive chondrogenic mucopolysaccharide staining were observed in each corresponding staining (Fig. 1b–d). Additionally, HUCMSC-specific markers were detected using flow cytometry. HUCMSCs were positive for the typical stem cell surface markers CD29, CD105, and CD44, but were negative for the hematopoietic lineage markers CD34 and CD45 (Fig. 1e and f).

### Characterisation of human articular chondrocyte-derived EVs

**Fig. 2:**
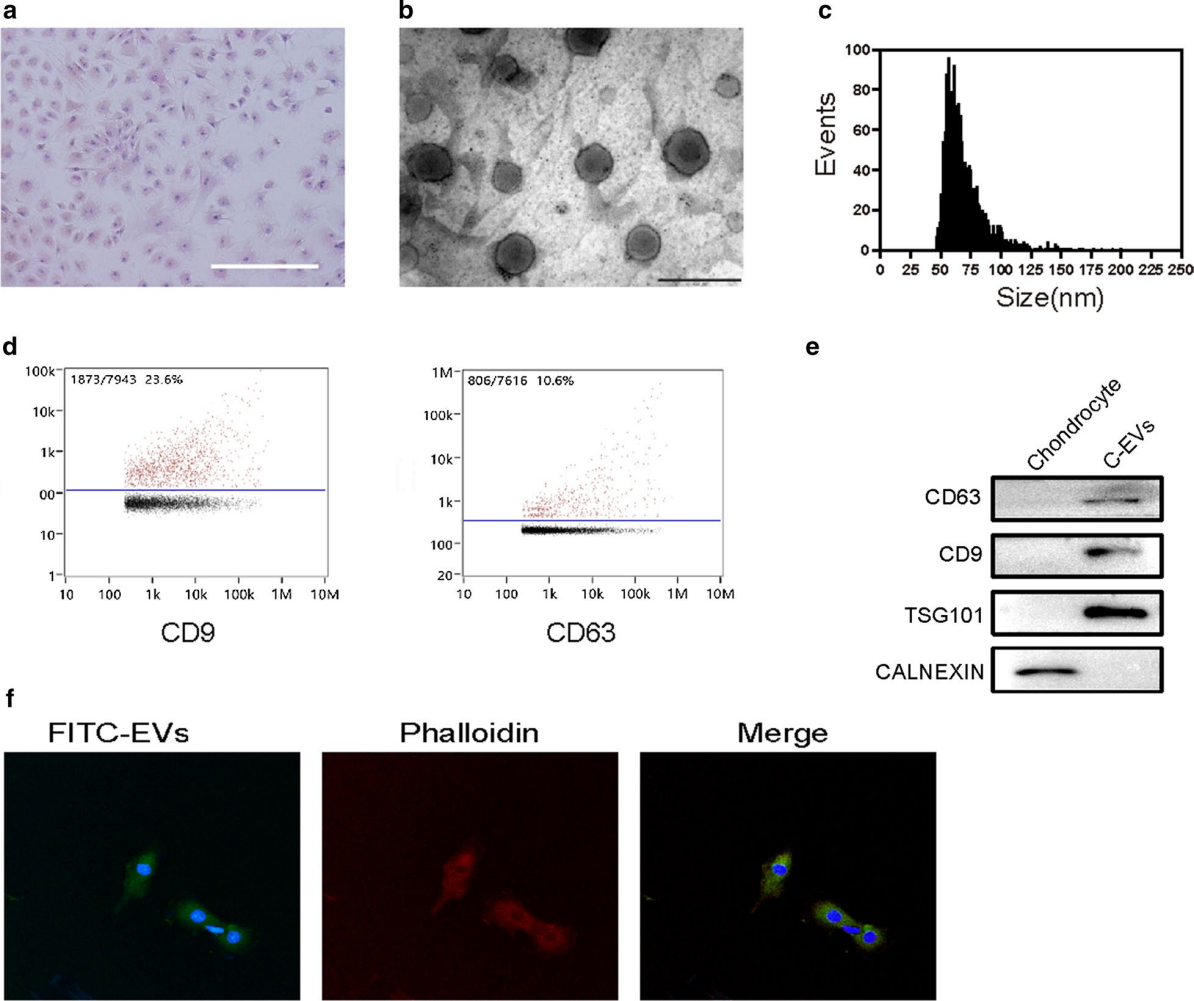
Characterization of human C-EVs. **a** The morphology of chondrocytes was observed using haematoxylin–eosin staining. Scale bar = 500 μm. **b** The morphology of C-EVs was analysed by transmission electron microscopy. Scale bar = 200 nm. **c** The particle size distribution of C-EVs was measured using a flow nano analyser. **d** The expression of CD9 and CD63 in C-EVs was measured using a flow nano analyser. **e** Western blotting analysis of the protein levels of CD63, CD9, TSG101, and calnexin in C-EVs and chondrocytes. **f** The representative immunofluorescence photomicrograph for cellular uptake of FITC-labelled C-EVs (green) was obtained using confocal microscopy. Scale bar = 50 μm

To obtain articular chondrocyte-derived EVs, we first isolated and cultured human articular chondrocytes, which were observed that the cobblestone-like morphology (Fig. 2a). EVs were isolated from the conditioned medium of chondrocytes. Transmission electron microscopy (TEM) showed the spheroid morphology of the EVs purified from chondrocytes (Fig. 2b). To check the particle size of the EVs, we performed Flow Nano Analysis (NanoFCM) and confirmed that the size was within 40–150 nm in diameter (Fig. 2c). The EV-related markers CD9 and CD63 were detected by using NanoFCM (Fig. 2d). Further, the levels of CD63, CD9, TSG101 and CALNEXIN in C-EVs and chondrocytes were analyzed by Western blotting. As shown in Fig. 2e, CD63, CD9 and TSG101 proteins were expressed in C-EXO, but not CALNEXIN. Collectively, these results indicate that EVs derived from human articular chondrocytes were successfully isolated and identified. Moreover, to investigate the feasibility of using C- EVs for the treatment of cartilage defect, we examined cellular uptake of C-EVs by HUCMSCs in vitro. After incubation of HUCMSCs with FITC-labelled C-EVs for 12 h, fluorescence microscopy images revealed that FITC-labelled C-EVs were present in the cytoplasm of HUCMSCs, confirming that C-EVs were internalised successfully by HUCMSCs (Fig. 2f).

### C-EVs accelerate the proliferation and migration of HUCMSCs

**Fig. 3:**
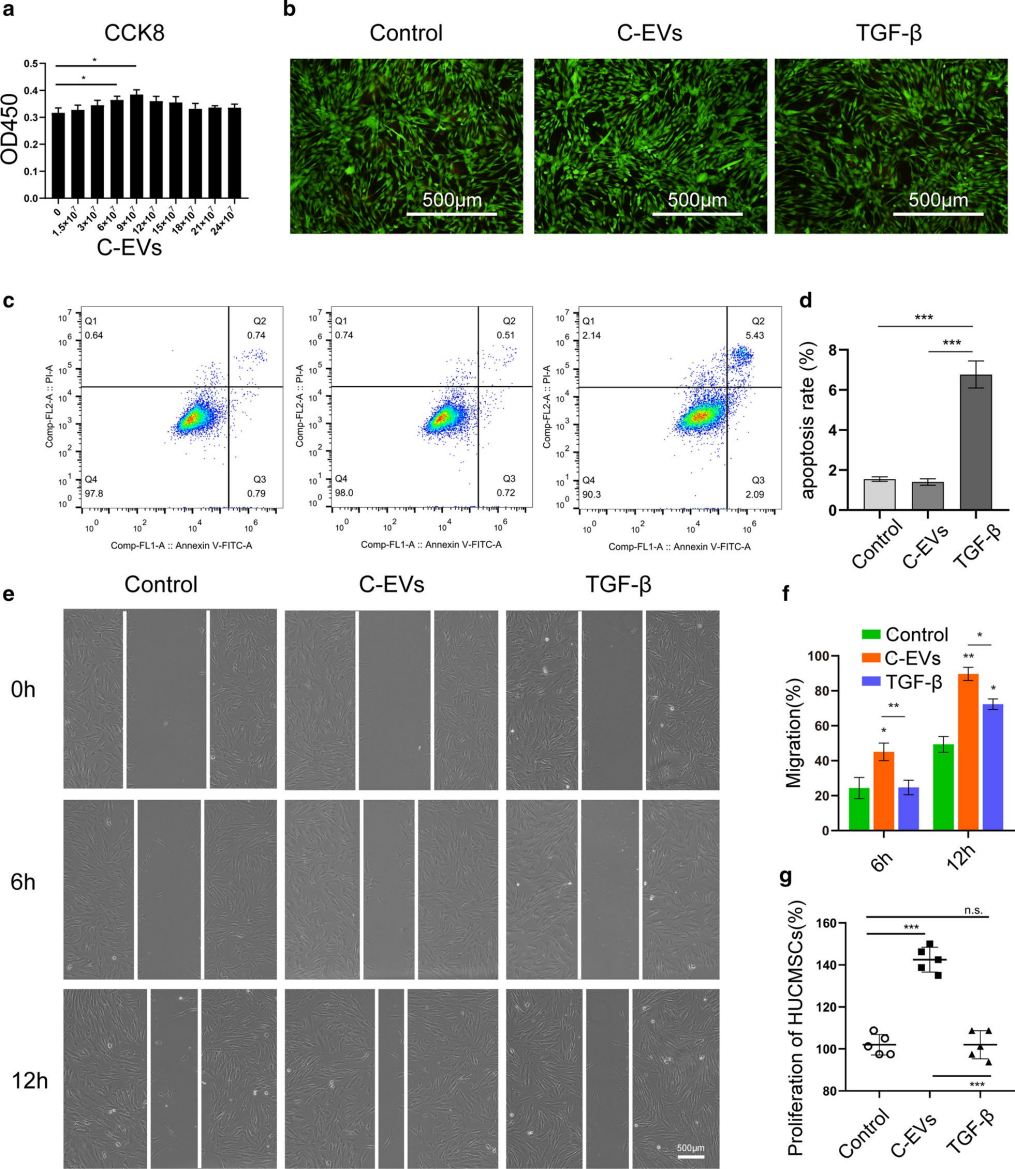
The C-EVs accelerate the proliferation and migration of HUCMSCs. **a** The cytotoxicity of HUCMSCs was detected after treatment with C-EVs at different doses using the CCK-8 assay. The quantitative data are presented as the means ± standard deviations (SD) of three independent experiments. *p < 0.05. **b** Representative images of the live/dead cell staining of HUCMSCs after treatment with PBS, C-EVs, or TGF-β were obtained by fluorescence microscopy. Scale bar = 500 μm. **c** The apoptotic index was determined using flow cytometry analysis. (D) Quantitative estimation of the proportion of apoptotic cells from (C), the quantitative data are presented as the mean ± SD of three independent experiments. *p < 0.05, **p < 0.01. **e** The light microscopic images from the scratch wound assay. Scale bar = 500 μm. **f** The quantitative analysis of HUCMSC migration at 6 h and 12 h, the quantitative data are presented as mean ± SD of three independent experiments. *p < 0.05, **p < 0.01. **g** The counts of HUCMSCs in different groups at 12 h. *n.s*. no significant difference, ***p < 0.001

To assess the effect of C-EVs on the proliferation of HUCMSCs, we evaluated the viability of HUCMSCs cultured with various doses of C-EVs. According to the result of CCK8 analysis, C-EVs promoted HUCMSC growth in a dose-dependent manner. Among the concentrations, the corresponding productivity of HUCMSCs appeared at the highest level at 9 × 10^7^ particles per mL (Fig. 3a). Thus, this concentration was selected for the following experiments. The results of both the live/dead cells viability assay and flow cytometry analysis showed that the proportion of live cells/dead cells in the C-EVs group was comparable with the control group. However, the proportion of apoptotic cells was higher in the TGF-β group compared with the C-EVs group, indicating that C-EVs stimulation did not lead to HUCMSCs apoptosis in vitro (Fig. 3b–d). To analyse whether the migratory ability of HUCMSCs was affected by C-EVs, HUCMSCs were incubated with C-EVs for 6 and 12 h. The results of the Scratch wound healing experiment showed that both C-EVs and TGF-β significantly enhanced the migratory capacity of HUCMSCs relative to the control. Notably, C-EVs exhibited a more positive effect on the motility of HUCMSCs than TGF-β did at every time point (Fig. 3e–g). These results implied that C-EVs promote the proliferation of HUCMSCs, although the underlying mechanism needs to be clarified further.

### C-EVs promote the chondrogenic differentiation of HUCMSCs

**Fig. 4:**
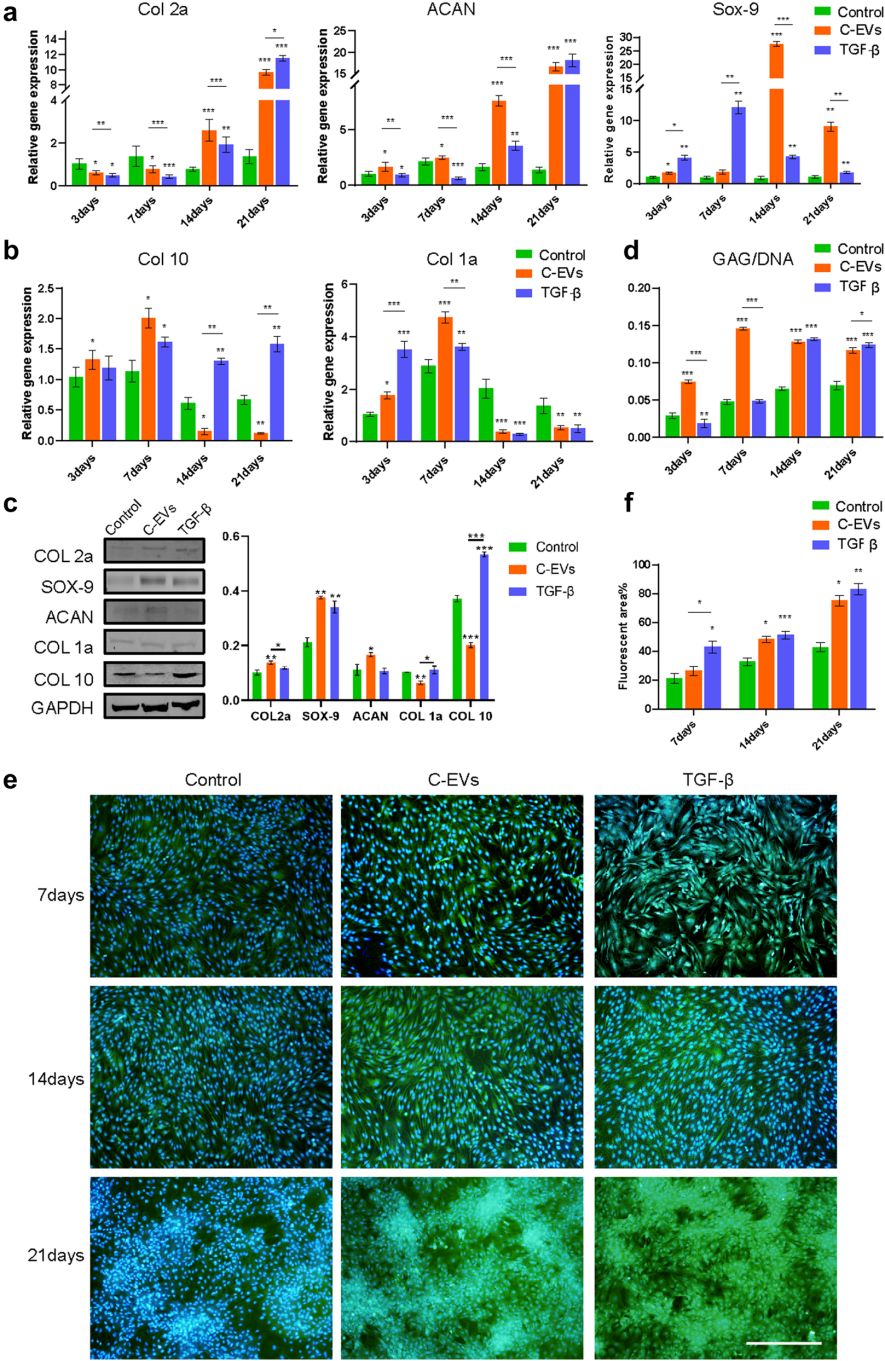
C-EVs promote the chondrogenic differentiation of HUCMSCs. The expression levels of chondrocyte-specific markers Col2a, ACAN, and Sox9 (**a**), the hypertrophic cartilage marker Col10, and the fibrocartilage marker Col1a (**b**) in C-EVs-stimulated HUCMSCs were determined using RT-PCR analysis. These data are presented as the mean ± SD of three independent experiments. *p < 0.05, **p < 0.01, ***p < 0.001. **c** The amount of COL2A, SOX9, ACAN, COL1A, and COL10 protein was determined using western blot analysis of HUCMSCs 21 days after treatment with PBS, C-EVs, or TGF-β. GAPDH was used as a loading control. **d** The quantification of DNA and GAG was performed using biochemical assays, and the ratio of GAG/DNA was calculated. HUCMSCs were cultured with PBS, C-EVs, or TGF-β for 3, 7, 14, and 21 days. These data are presented as the mean ± SD of three independent experiments. *p < 0.05, **p < 0.01, ***p < 0.001. **e** Immunofluorescence staining for COL2A in HUCMSCs treated with or without C-EVs in vitro. Scale bar = 500 μm. **f** Quantitative results of immunofluorescence in **e**, *p < 0.05, **p < 0.01, ***p < 0.001

To investigate a possible role of C-EVs in chondrogenic differentiation, HUCMSCs were allowed to undergo chondrogenic differentiation in the absence or presence of C-EVs. TGF-β was used as a positive control. As shown in Fig. 4a, we observed that the expression of the cartilage-specific marker Col2a was slightly downregulated during the initial period. Nevertheless, after prolonged stimulation of C-EVs, the expression of Col2a dramatically increased at 14 and 21 days, and the expression of Sox9 and ACAN, known to be involved in chondrogenesis, was significantly increased at each time point after C-EVs treatment compared with the negative control, as demonstrated by RT-PCR analysis. The upregulation of these genes was comparable with that in the TGF-β induction group. Of note is that the expression of Col1a, a fibrocartilage marker, was also elevated at early stages, but significantly decreased at later stages (14 days and 21 days) in both the C-EVs and TGF-β group compared with controls. In addition, we also examined the expression of the hypertrophic cartilage-enriched marker Col10, and found its expression to be equal to Col1a at 3 and 7 days.

In contrast, it was heavily downregulated in the C-EVs group compared with the untreated group. Still, it maintained a relatively high level in the TGF-β group at 14 and 21 days (Fig. 4b). Western blot analysis also showed COL2A, SOX9, and ACAN protein expressions were significantly upregulated, whereas COL1A and COL10 protein expressions were downregulated in the C-EVs group. Notably, the expression of COL10 protein also remained at high levels in the TGF-β group (Fig. 4c). Measurements of glycosaminoglycans (GAG) were carried out using the established DMMB (1,9-dimethylmethylene blue) assay. The level of GAG accumulation was normalised by DNA content, which served as the cell number control. As expected, GAG production was increased in both the C-EVs and TGF-β groups; however, the timepoint at which it was increased was earlier for the C-EVs group (3 days) than the TGF-β group (Fig. 4d and Additional file 1: Fig. S1). Furthermore, the results of immunofluorescence staining for COL2A demonstrated that COL2A expression was more elevated than the negative control at 21 days in both the C-EVs and TGF-β groups (Fig. 4e and f), which is consistent with the RT-PCR results. Taken together, these data strongly suggest that C-EVs contribute to the chondrogenic differentiation of HUCMSCs, and their influence is more beneficial than TGF-β owing to the ability of C-EVs to maintain the phenotypic stability of chondrocytes.

### C-EVs promote chondrogenesis by activating autophagy in HUCMSCs

**Fig. 5:**
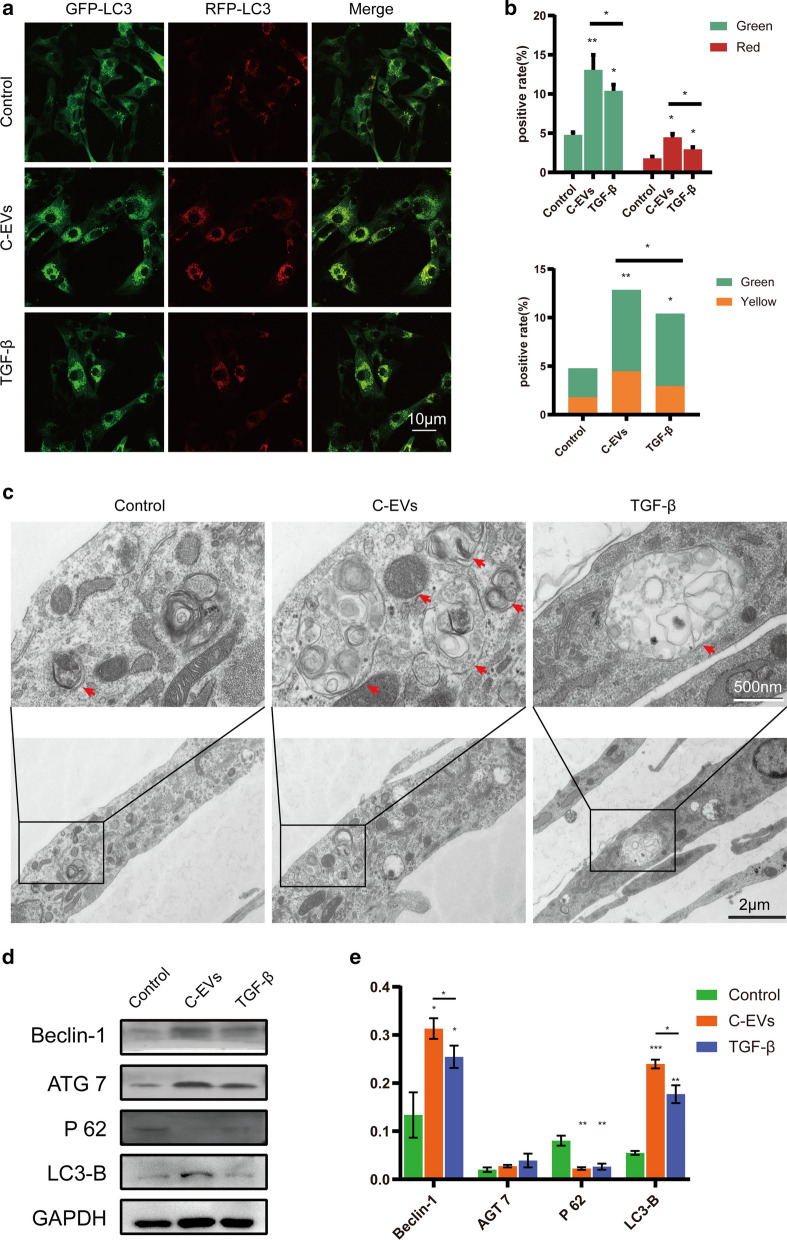
C-EVs treatment induces the activation of autophagy in HUCMSCs. **a** The autophagic flux of mRFP-GFP-LC3-transfected HUCMSCs was revealed using laser confocal microscopy. Autophagosomes are labelled by red and green fluorescence (yellow spots), whereas autophagic lysosomes are labelled by red fluorescence (red spots). **b** The percentage of fluorescence from (**a**) were quantified and presented as the mean ± SD of three independent experiments. *p < 0.05, **p < 0.01, ***p < 0.001. **c** The formation of autophagic vacuoles was observed by transmission electron microscopy. Boxed regions are enlarged and shown in insets. Red arrows indicate autophagic vacuoles. Scale bar = 2 μm. **d** HUCMSCs and C-EVs were incubated for 14 days, and then western blot analysis of autophagy-associated protein levels was performed. **e** Beclin-1, ATG7, P62, and LC3-B protein expressions were quantified and referred to GAPDH, then presented as the mean ± SD for each group. *p < 0.05, **p < 0.01, ***p < 0.001

Given that C-EVs promoted the chondrogenic differentiation of HUCMSCs and that autophagy plays a significant role in cell differentiation [22, 23], we investigated whether autophagy activation will occur during the chondrogenic differentiation of HUCMSCs mediated by C-EVs. First, HUCMSCs were transfected with mRFP-GFP-LC3 virus and cultured with or without C-EVs, and autophagic flux was observed using laser confocal microscopy. This fluorescent reporter is used to monitor LC3 flux based on different pH stability of green and red fluorescent proteins. The lysosome can quench the fluorescent signal of GFP in the acidic environment, whereas the RFP fluorescence signal persists under these environments. In green/red merged images, yellow puncta (RFP+GFP+) indicate autophagosomes, while red puncta (RFP+GFP-) indicate autolysosomes. Autophagic flux is increased when both yellow and red puncta are increased in cells [24]. As shown in Fig. 5a, there were more yellow and red fluorescence dots in the C-EVs group than in the TGF-β and negative control groups (Fig. 5a and b), indicating a higher number of autophagosomes in the C-EVs-treated HUCMSCs. Moreover, the appearance of autophagosomes was confirmed in C-EVs- or TGF-β-treated HUCMSCs by transmission electron microscopy (TEM). The C-EVs group showed more autophagosomes than the other groups (Fig. 5c), which is in line with the results of laser confocal microscopy. Further western blotting results showed that in the C-EVs group, the expression levels of HUCMSC autophagy of the positively correlated proteins Beclin-1, ATG7, and LC3-B were increased. In contrast, the expression level of the negatively correlated protein P62 was decreased (Fig. 5d and e). Thus, C-EVs stimulation induced the chondrogenic differentiation of HUCMSCs by activating autophagy in vitro.

### C-EVs promote cartilage repair during HUCMSC-mediated cartilage regeneration in vivo

**Fig. 6:**
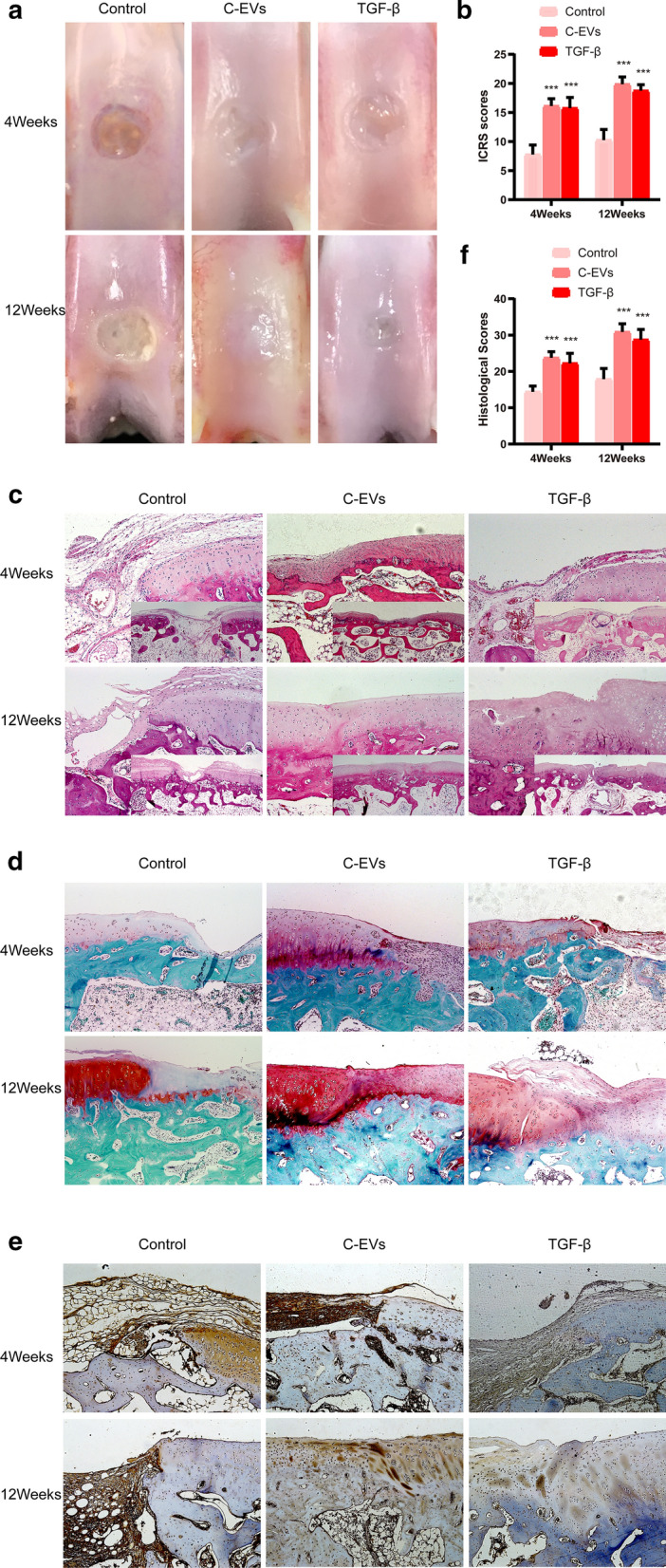
The C-EVs improve the therapeutic effect of HUCMSCs on articular cartilage repair in the rabbit model. **a** Gross morphology of repaired cartilage of the control group, The C-EVs group, and the TGF-β group at 4 weeks and 12 weeks post-surgery. **b** Macroscopic scores of regenerative tissues from the control group, the C-EVs group, and the TGF-β group. n = 12. ***p < 0.001. **c** H&E staining of repaired cartilage from the control group, the C-EVs group, and the TGF-β group. **d** Safranin O-fast green staining of repaired cartilage from the control group, the C-EVs group, and the TGF-β group. **e** Immunohistochemical staining of collagen type II in repaired cartilage from the control group, the C-EVs group, and the TGF-β group. **f** Histological scores of regenerative tissues from each group. n = 12. ***p < 0.001

To further test the cartilage repair efficacy of C-EVs, we generated an articular cartilage defect model in rabbits. None of the rabbits displayed signs of post-operative infection or lameness during the 12-week post-transplantation period. Repair of articular cartilage defects was assessed at 4 and 12 weeks after HUCMSC implantation surgery. As shown in Fig. 6a, 4 weeks after transplantation, the macroscopic appearance of all three groups displayed recognisably regenerated tissue. Interestingly, in a comparison of all groups, the regenerated tissues in the C-EVs and TGF-β groups were better than the control group. In contrast, the C-EVs group had more hyaline-like cartilaginous repair tissue, and the concave surface of the regenerated tissue was smaller than that of the TGF-β group. After 12 weeks, there was still a noticeable hole in the tissue surface of the control group, the cartilage defect was nearly devoid of regenerative tissue, with only amorphous soft fibres observed in the central area of the defect. The repaired tissues of the treatment groups were almost identical to healthy cartilage tissue. Notably, the newly formed tissues in the C-EVs groups had a smooth white appearance and fused with the surrounding healthy cartilage; its surface topography was more similar to that of the host cartilage compared with that of the TGF-β group, whose regenerated tissue surfaces within the defect were fairly rough (Fig. 6a). Further quantitative assessment of repaired tissue showed that both the C-EVs group and the TGF-β group had significantly higher ICRS scores (mean ± SD of 16.125 ± 1.25, mean ± SD of 15.75 ± 1.83, respectively) than the control group (mean ± SD of 7.75 ± 1.67) (p<0.05) (Fig. 6b). In addition, the gross morphological findings were supported by histological evidence. H&E staining showed that only a small amount of fibrous tissue was observed in the periphery of the defect and lacked hyaline-like cartilaginous tissue in the control group, suggesting limited intrinsic repair capability.

In contrast, regenerated tissues in the C-EVs and TGF-β groups presented a smooth surface like hyaline cartilage with regular cellular tissue, which was more abundant and thicker than those in the control group (Fig. 6c). Safranin O-Fast Green staining demonstrated the changes in the quantity of proteoglycan in the cartilage matrix. As shown in Fig. 6d, the content of proteoglycan in the C-EVs group is relatively large and evenly distributed in the cartilage matrix at 12 weeks. The cartilage-like tissues entirely occupied the defects, the regenerated tissue was similar in colour and texture to native cartilage, and even the formation of the cartilage matrix was closer to healthy cartilage.

**Fig. 7:**
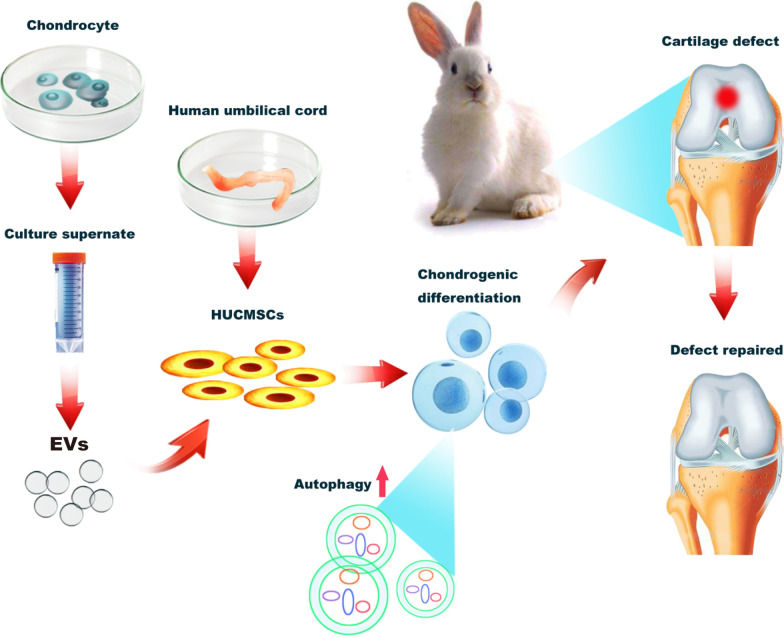
The graphic illustration for C-EVs promoting chondrogenic differentiation of HUCMSCs and enhancing cartilage repair in vivo, meanwhile, autophagy was activated during the induction of HUCMSCs differentiation

In contrast, the production of proteoglycan was lower in the TGF-β group compared with the C-EVs group. In addition, at week 12, positive expression of collagen type II was found in the C-EVs group and TGF-β group, but not in the control group, and the positive expression of collagen type II in the C-EVs group was more similar to healthy tissues (Fig. 6e). Quantitatively, the mean score of the control group was significantly lower than that of the C-EVs group and the TGF-β group. Among these groups, C-EVs treatment exhibited the best restorative effect (Fig. 6f). Taken together, these results suggested that C-EVs can promote cartilage defect repair in HUCMSC-mediated cartilage regeneration (Fig. 7).

## Discussion

In this study, we demonstrated that chondrocyte-derived exosomes could promote chondrogenic differentiation of HUCMSCs by activating autophagy in vitro and improve the effect of HUCMSC-mediated cartilage repair in vivo.

During the repair of articular cartilage defects using the stem cells, the microecological environment of the tissue should have the same characteristics as the host cartilage. One of the main niche cell types in the joint is chondrocytes, which play an important role in maintaining the microenvironment in the joint cavity. In our study, we found that C-EVs could promote the chondrogenesis of HUCMSCs and exert a therapeutic effect on cartilage defects, suggesting that the C-EVs provide a pleasant micro-ecological environment for cartilage repair. This finding is consistent with previous studies that EVs derived from mature chondrocytes can promote the formation of ectopic cartilage in the subcutaneous environment of cartilage progenitor cells [18]. EVs, as important paracrine components, are involved in maintaining normal physiological functions, mediating inter-cell communication, and inducing changes in cell functions and processes by delivering various types of bioactive microRNAs, proteins, and unique gene products [9, 10, 25]. These microRNAs might serve as vital inducers of HUCMSCs differentiation into chondrocytes, such as the miR-92a-3p, miR-95-5p, miR-320c, and miR-135b, which can regulate cartilage development and homeostasis [26–29]. These findings suggest that the active molecules in EVs may be an important factor underlying their ability to promote chondrogenesis of HUCMSCs. Thus, to a great extent, our results explain the research finding that the co-culture of chondrocytes induces chondrogenesis of mesenchymal stem cells [30, 31], which might be due to the stimulation of chondrocyte-secreted EVs. At present, the exact role of C-EVs in cartilage repair remains unclear, and we suspect that microRNAs or proteins related to autophagy may play a key role. The contents of C-EVs are complex, and hence future studies may be needed to analyse further the components and mechanisms involved in cartilage repair for these EVs. Interestingly, EVs derived from osteoblast can promote osteogenic differentiation of stem cells in an adipogenic medium environment [16]. These results combined with our findings further support the biological theory that EVs retain the original biological activities of homologous cells.

Our research also revealed that C-EVs enhance *Sox9* and *Col2a1* expression, particularly in the late stage of chondrogenic differentiation. Moreover, the upregulated levels of these genes were comparable with TGF-β upregulation. It is worth noting that the expression of Col10, a hypertrophic cartilage-related marker, was upregulated in the TGF-β group. These findings indicated that although TGF-β could promote HUCMSC differentiation into chondrocytes, it was prone to inducing hypertrophic differentiation. This result is in accord with the results of others who reported that TGF-β as a main transforming growth factor causes cartilage hypertrophy during the process of inducing cartilage differentiation [5].

Interestingly, we found that compared with TGF-β, C-EVs had a superior effect in chondrogenic specificity, as evidenced by remarkably downregulated expression of Col1a (a fibrocartilage marker) and Col10 (a hypertrophic cartilage marker), indicating that C-EVs can better prevent fibrogenic and hypertrophic differentiation by maintaining the chondrocyte phenotype. This notion of chondrogenesis is further supported by the observations that C-EVs administration accelerated the healing process of cartilage defects and maintained the characteristics of hyaline cartilage, as evidenced by histological findings and macroscopic appearance, which displayed more effective cartilage repair than TGF-β did in a rabbit cartilage defect model. Therefore, C-EVs possess a robust ability to repair cartilage and cause less hypertrophy, which bypasses the shortcomings of TGF-β.

In our studies, we also observed that the chondrogenic differentiation of HUCMSCs mediated by C-EVs was accompanied by the activation of autophagy, suggesting that autophagy is probably an essential process during chondrogenic differentiation mediated by C-EVs. Direct interaction of autophagy and maintenance of chondrocytes has been demonstrated in other studies where autophagy protects chondrocytes from glucocorticoid-induced apoptosis [32, 33], confirming the boosting effect of autophagy on the survival biosynthetic function of chondrocytes. Interestingly, other researchers have shown that autophagy regulates the formation of articular cartilage vesicles in primary articular chondrocytes [34], indicating that there may be mutual regulation between vesicles and autophagy. Notably, our studies showed no evidence of cell death during the induction of autophagy within the periods in which C-EVs induced chondrogenic differentiation of HUCMSCs. Besides, we found that C-EVs could also significantly stimulate HUCMSCs migration and proliferation, probably by modulating the cell cycle, although the underlying mechanism needs further clarification. Further studies are required to evaluate the possible use of C-EVs to stimulate autophagy during chondrogenic differentiation of HUCMSCs and as a therapeutic agent in cartilage defect repair.

## Conclusion

In summary, this study characterised C-EVs from the perspective of cartilage differentiation in HUCMSCs. Our results demonstrate for the first time that human C-EVs strongly promote differentiation of HUCMSCs into chondrocytes and significantly activate autophagy during the process. These studies shed new light on the development of potential C-EVs therapies that exploit chondrogenesis inductive capabilities.

## Materials and methods

### Isolation and culture of HUCMSCs

The sampling scheme of human umbilical cord tissue was approved by the Institutional Review Board of The First Affiliated Hospital of Guangxi Medical University. Signed informed consent was obtained from all participants for this study. The isolation of HUCMSCs from the Wharton^’^s jelly of the umbilical cords was described previously [35] with minor modifications. In brief, umbilical cords were obtained from full-term delivery patients by caesarean section at The First Affiliated Hospital of Guangxi Medical University. The Wharton^’^s jelly was isolated and cut into 1–2-mm [3] pieces, then the growth medium was added and cultured at 37 °C in a 5% CO_2_ humidified incubator. The growth medium was composed of high-glucose Dulbecco’s modified Eagle’s medium (DMEM; Gibco, USA) and 10% foetal bovine serum (FBS; Gibco, USA) was refreshed every 3 days. After the first subculture, the cells were passaged at a ratio of 1:5 when they reached near 90% confluence. All HUCMSCs undergoing further analysis were used at passages 3–5 in this study.

### The osteogenic, adipogenic and chondrogenic differentiation capacity of isolated

HUMSCs were assessed using Osteogenic-Differentiation Medium, Adipogenic-Differentiation Medium, and Complete Chondrogenic Medium (Cyagen Biosciences, Santa Clara, CA, USA). At the end of the incubation period, the differentiation of HUCMSCs was detected by three kinds of staining. Alizarin red solution (Solarbio, Beijing, China), oil red-O staining (Solarbio, Beijing, China), and alcian blue staining (Solarbio, Beijing, China) were used to detect osteogenic differentiation, lipogenic differentiation, and chondrogenic differentiation according to manufacturer’s instructions, respectively.

### Isolation and culture of mature chondrocytes

Primary chondrocytes were harvested from patients (n = 13, average age: 10.8 months, range 6–15 months, male: 7, female: 6) who underwent polydactyly surgery at The First Affiliated Hospital of Guangxi Medical University. Cartilage tissues were obtained from knuckle cartilage and minced into pieces. And then, the cartilage pieces were digested using a 2 mg/mL collagenase type II solution (Sigma, USA) at 37 °C for 3 h after treatment with 0.25% trypsin (Solarbio, Beijing, China) for 30 min as described previously [6]. Chondrocytes were cultivated in DMEM culture medium containing 10% foetal bovine serum (Gibco, USA) and 1% penicillin/streptomycin (Solarbio, Beijing, China) at 37 °C in a 5% CO_2_ humidified incubator. The culture medium was replaced by fresh medium every 3 days. The chondrocytes were re-plated at a ratio of 1:4 when the cells achieved near 100% confluence. All the chondrocytes at passages 3–4 that remained oval or polygonal in shape were used in our studies.

### Extraction and identification of C-EVs

The C-EVs were extracted by differential centrifugation, as described previously, with some minor modifications [36, 37]. Briefly, the culture medium was collected after chondrocytes were cultured with serum-free medium for 48 h, centrifuged at 1000×*g* at 4 °C for 15 min to remove dead cells, and then centrifuged at 10,000×*g* for 30 min to remove cell debris and microparticles. The supernatant was collected and transferred to ultracentrifuge tubes (Beckman Coulter) and ultra-centrifuged at 100,000×*g* for 1.5 h using a Thermo Scientific Sorvall WX UltraSeries Centrifuge with an AT-50 rotor. The pellets were collected and washed with PBS by centrifugation at 100,000×*g* for 1.5 h. All centrifugation procedures were carried out at 4 °C. The purified C-EVs were resuspended in PBS and stored at − 80 °C. The C-EVs were identified by transmission electron microscopy (TEM, H-800, Hitachi, Tokyo, Japan) and a Flow Nano Analyzer (NanoFCM, Xiamen Fuliu Biological Technology Co., China).

### Cellular uptake of C-EVs

The C-EVs were first labelled with FITC-CD9 according to the manufacturer’s instructions (BD Biosciences, USA). Briefly, 200 μL of the cell-labelling solution was added to 500 μL of an EVs suspension (1.77E+10 Particles/mL) and incubated at 37 °C for 30 min. Subsequently, the mixture was washed by PBS twice to remove the free dye, and then the sediment was suspended with 500 μL of PBS. The HUCMSCs were incubated with labelled C-EVs at 37 °C for 12 h. The HUCMSCs were then fixed with 4% paraformaldehyde at room temperature for 20 min after washing with PBS. Next, cells were dyed with phalloidin and DAPI. Confocal images were sequentially acquired by confocal microscopy (TCS SP8, Leica, Germany).

### Cell proliferation assay

Cell counting kit-8 (CCK8, Beyotime, Biotechnology, Haimen, China) analysis was used to measure the proliferation of cells. The HUCMSCs were cultured in a 96-well plate. After the cells reached 70–80% confluence, the culture medium was substituted with 90 μL of a fresh medium comprising various concentrations of C-EVs and cultured for 24 h. After that, 10 μL of CCK-8 solution was added to each well, and the optical density (OD) of the culture medium was measured at 450 nm using a microplate reader (Thermo Scientific, USA) after incubation at 37 °C for 4 h following a previously reported study [38].

### Cell viability assay

A live/dead cell viability assay was used to measure the viability of HUCMSCs. According to the manufacturer’s instructions (Invitrogen, Carlsbad, CA, USA), the staining solution was prepared as 0.5% calcein–acetoxymethyl (calcein-AM) and 2% propidium iodide (PI) in PBS. Then, the treated or untreated HUCMSCs were washed three times with PBS and stained with the staining solution at 37 °C for 5 min in the dark. Images were captured by the inverted fluorescence microscope (BX53, Olympus, Tokyo, Japan).

### Migration assay

The migration of C-EVs-treated HUCMSCs was evaluated using scratch wound assay. HUCMSCs were plated in a 12-well plate (2 × 10^5^ cells/well, three replicates per group) and cultured in a 37 °C humidified incubator with 5% CO_2_ until cell fusion. The confluent monolayer of cells was scratched using a 10-μL micropipette tip and then washed with culture medium three times to remove shed cells. After that, the HUCMSCs were cultured in a serum-free medium containing C-EVs, TGF-β (10 ng/mL), or control medium. The images were obtained at the same position at 0 h, 6 h, and 12 h post-wounding. Scratched areas were investigated via Image-J software (National Institutes of Health, Bethesda, MD, USA).

### Flow cytometry

Cells of each group were harvested into two Eppendorf tubes (sample tubes and negative tubes) and centrifuged at 1200 rpm for 5 min, and then the pellet was resuspended by adding 50 μL of binding buffer. Followed by the dyes (FITC Annexin V Apoptosis Detection Kit, BD Biosciences) was added in sample tubes but not negative tubes. After the solution was shaken gently and mixed, it was incubated for 15 min at room temperature under the dark environment. Then the samples were treated with 200 μL of binding buffer and analysed by flow cytometry (BD AccuriTMC6 PLUS, BD Biosciences). For C-EVs detection, fluorescently labelled antibody: human CD9 FITC (2 μL, BD Pharmingen™, Catalog No. 555371), human CD63 FITC (2 μL, BD Pharmingen™, Catalog No. 550759) were added into 50 μL of C-EVs. After incubation at 37 °C for 30 min, labelled C-EVs were washed twice, and then the particles were resuspended in PBS and tested using a Flow Nano Analyzer (NanoFCM, Xiamen Fuliu Biological Technology Co., China).

### Autophagy detection

The observation of autophagy was conducted by double-labelled mRFP-GFP-LC3 adenovirus (Shanghai Genechem Co., China) transfection. First, the HUCMSCs were cultured for 2 days, and then mRFP-GFP-LC3 lentivirus was introduced into HUCMSCs according to the manufacturer’s protocol. After that, these cells were treated with PBS, C-EVs, and TGF-β, respectively, for 48 h. The formation of autolysosomes was detected and analysed using laser confocal microscopy (TCS SP8, Leica, Germany). Autophagic bodies are represented by yellow spots and autophagic lysosomes by red spots. In addition, cells from different groups were sectioned, and autophagosomes were observed using transmission electron microscopy (TEM, H-800, Hitachi, Tokyo, Japan).

### Real-time polymerase chain reactions

Total RNA was extracted from each group of cells using a total RNA isolation kit (Tiangen Biotechnology, Beijing, China) according to the manufacturer’s protocol. The quantity and purity of RNA were determined using Nanodrop (Thermo Scientific, USA). One thousand ng of total RNA samples were used to synthesise complementary DNA using a reverse transcription kit (Fermentas, Waltham, MA). Real-time polymerase chain reaction (PCR) was conducted using FastStart Universal SYBR Green Master Mix (Roche Company, Basel, Switzerland) and pre-designed primers for 45 cycles of 15 s at 95 °C and 1 min at 60 °C as reported previously [39]. The primer sequences of the genes are listed in Table 1. The expression level of each gene was normalised using glyceraldehyde -3-phosphate dehydrogenase (GAPDH). Each experiment was repeated three times. The target gene relative expression was calculated using the comparative method 2^−Δ^Ct.

**Table 1:** Primers for real-time polymerase chain reaction

Gene names	Forward primer	Reverse primer
Col2a	CCGTGCTCCTGCCGTTTC	CTGAGGCAGTCTTTCACGTCT
Sox9	AAGCTCTGGAGACTTCTGAACG	CGTTCTTCACCGACTTCCTCC
Acan	CTACACGCTACACCCTCGAC	ACGTCCTCACACCAGGAAAC
Col10	CGATACCAAATGCCCACAGG	ATGGTCCTCTCTCTCCTGGT
Col1a	GTTCAGCTTTGTGGACCTCCG	GCAGTTCTTGGTCTCGTCAC
GAPDH	GTCAAGGCTGAGAACGGGAA	AAATGAGCCCCAGCCTTCTC

Col2a: collagen type II; Sox9: SRY‐related high mobility group‐box gene 9; Acan: aggrecan; Col10: collagen type X; Col1a: collagen type I; GAPDH: glyceraldehyde‐3‐phosphate dehydrogenase

### Western blot analysis

The western blot analysis was performed as previously described [40]. Briefly, collected cells were washed with cold PBS and lysed in lysis RIPA buffer containing PMSF (1 mM phenylmethylsulfonyl fluoride). The extracted protein concentration was determined using a BCA protein assay kit (Beyotime, China). Furthermore, each sample with an equal amount of protein (70 μg) was added and separated on 10% SDS–polyacrylamide gel and then transferred to polyvinylidene fluoride membranes. Next, the membranes were incubated with an appropriate concentration of primary antibodies, which are as follows: CD63(1:1000, Abcam, Catalog No. ab134045), CD9(1:2000, Abcam, Catalog No. ab92726), TSG101(1:2000, Abcam, Catalog No. ab125011), CALNEXIN(1:1000, Abcam, Catalog No. ab22595), SOX9 (1:1000, CST, Catalog No. 82630), COL2A (1:1000, Abcam, Catalog No. ab185430), ACAN (1:100, Abcam, Catalog No. ab3778), COL1A (1:1000, Abcam, Catalog No. ab88147), COL10 (1:300, Abcam, Catalog No. ab58632), Beclin-1 (1:2000, Abcam, Catalog No. ab207612), ATG7 (1:1000, Cell Signaling Technology, Catalog No. 2631), P62 (1:1000, Cell Signaling Technology, Catalog No. 5114), LC3-B (1:2000, Abcam, Catalog No. ab18709) and GAPDH (1:5000, Abcam, Catalog No. ab8245). After washing with TBST at 37 °C, the membranes were incubated with a secondary antibody (1:15,000, LI-COR, USA). Images were acquired with the Odyssey infrared imaging system (LI-COR Biosciences, Lincoln, NE, USA).

### Establishment of animal models

All animal experiments included in this study were approved by The Animal Research Committee of the Guangxi Medical University. The surgical procedure for the rabbit model was performed as previously described [41]. Briefly, clinically healthy New Zealand white rabbits (either sex, 6-months-old, weighing 1–2 kg) were selected randomly for the study. General anaesthesia was administered as 2% pentobarbital sodium through the auricular vein, and the knee joint of the rabbit was exposed through the lateral parapatellar approach. A cartilaginous defect with a diameter of 4.0 mm and a depth of 3.0 mm was made in the medial side of each patella groove using a hand drill. Then, each animal received a local fill with masses of HUCMSCs (2 × 10^6^) in the defect area. The defects were treated as follows: (1) HUCMSCs, which were cultured with complete medium for 14 days (negative control group, n = 30 knees); (2) C-EVs-treated HUCMSCs: HUCMSCs were cultured with complete medium including C-EVs for 14 days, (C-EVs group, n = 30 knees); (3) TGF-β-treated HUCMSCs: HUCMSCs were cultured in chondrocyte-inducing medium with TGF-β for 14 days, which served as a positive control. (TGF-β group, n = 30 knees). Penicillin was administered for 3 days post-surgery. Euthanasia was performed with an overdose of pentobarbital sodium administered by intravenous (I.V.) injection at 4 or 12 weeks after surgery. The treated condyles were harvested for further analysis. The International Cartilage Repair Society (ICRS) scoring system was applied to assess the gross morphological phenotype of the cartilage defect repair in rabbit models [42].

### Histological examination

The osteochondral blocks containing repaired tissue were harvested, and specimens were decalcified with 14% ethylenediaminetetraacetic acid (EDTA) solution for six to eight weeks after fixation with 10% neutral buffered formalin. The tissue samples were embedded in paraffin and serially sectioned conventionally. The sections were stained with haematoxylin and eosin (H&E), safranin-O/fast green (Solarbio, Beijing, China) and immunohistochemical collagen type II stain (1:200, Bioss, Catalog No. bs-0709R) according to the standard protocols, then were observed using light microscopy (Olympus BX53, Tokyo, Japan). The results were scored by three qualified examiners who were blinded to the outcome of the cases according to the ICRS Visual Histological Assessment Scale [43].

### Immunofluorescence analysis

Immunofluorescence examination was performed as previously described [40]. For cell immunofluorescence preparation, planted cells were fixed by 4% paraformaldehyde, successively treated with 3% H_2_O_2_ for 10 min and blocked with goat serum at room temperature for 10 min. The primary antibody COL2A was used to detect changes in chondrogenic differentiation. Samples were incubated with anti-COL2A antibody (1:200, Boster, China) at 37 °C for 3 to 4 h, followed by incubation with the secondary antibody (anti-rabbit antibody, 1:50, Boster, China) at 37 °C for 45 min, the nuclei were counterstained with 4′, 6-diamidino-2-phenylindole (DAPI; Boster) for 5 min. Images were obtained sequentially using a fluorescence microscope (Olympus BX53, Tokyo, Japan).

### Statistical analysis

All data collected in this study are analysed in SPSS (SPSS v22.0; IBM, Armonk, NY). The statistical significance between the two groups was evaluated using a one-way analysis of variance with a t-test. A *P* < 0.05 was considered a statistically significant difference.

## Supplementary information

**Additional file 1: Fig. S1.** The DNA and GAG contents were stained for the Hoechst 33258 and dimethyl methylene blue dye binding assays, respectively, after 3, 7, 14, and 21 days of HUCMSCs treatment with negative control, C-EVs, and TGF-β. The absorbances were measured to quantify the contents of DNA (A) and GAG (B). These data are presented as the mean ± SD of three independent experiments. *p < 0.05, **p < 0.01, ***p < 0.001.

## Data Availability

The data supporting the findings of this study are available from the corresponding author upon reasonable request.
