# Supplementary figures and images for: Articular chondrocyte-derived extracellular vesicles promote cartilage differentiation of human umbilical cord mesenchymal stem cells by activation of autophagy
Source: J Nanobiotechnology. 2020 Nov 9;18:163. doi: 10.1186/s12951-020-00708-0 (PMC7653755; doi:10.1186/s12951-020-00708-0)

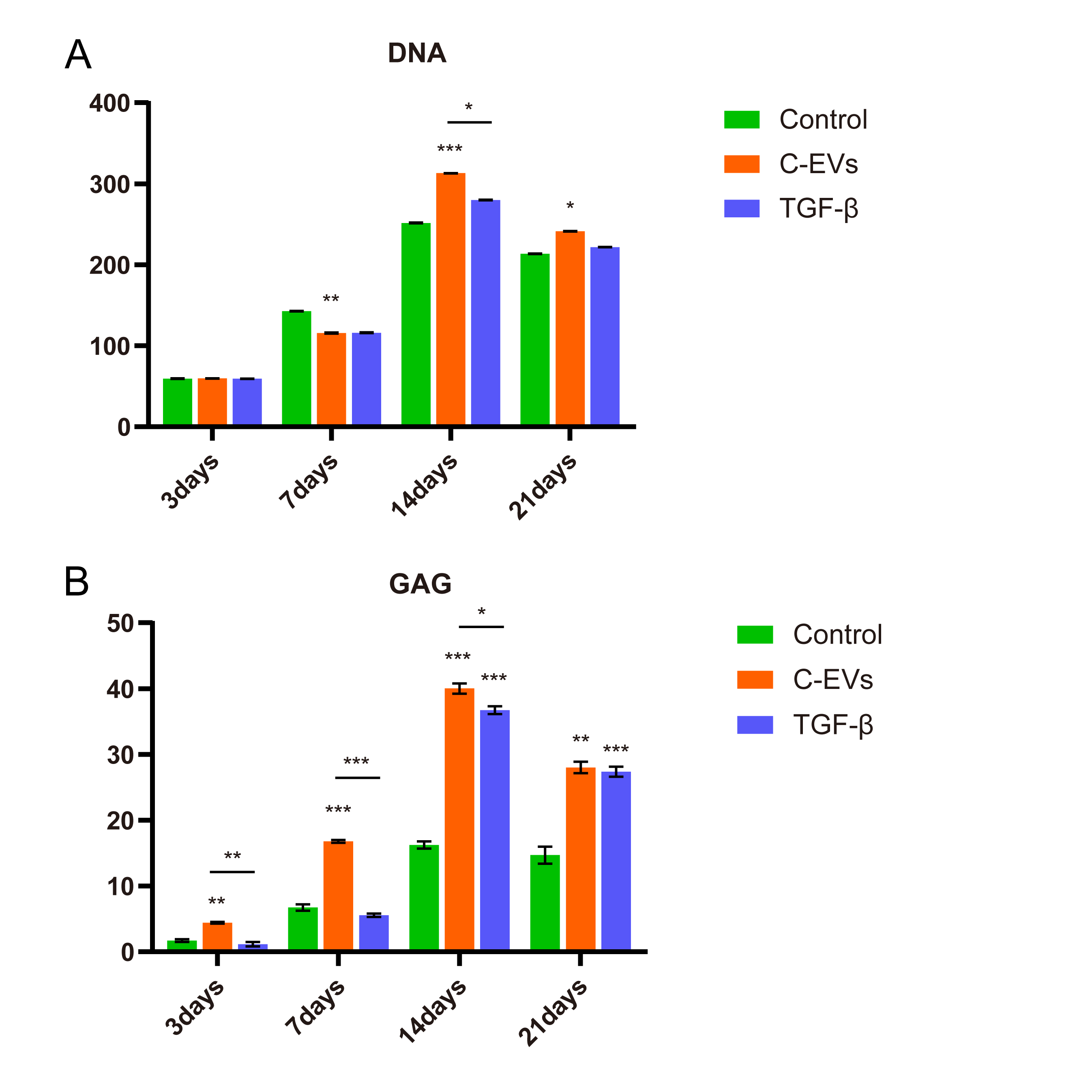

Supplement: Supplementary file 1 — Additional file 1: Fig. S1. The DNA and GAG contents were stained for the Hoechst 33258 and dimethyl methylene blue dye binding assays, respectively, after 3, 7, 14, and 21 days of HUCMSCs treatment with negative control, C-EVs, and TGF-β. The absorbances were measured to quantify the contents of DNA (A) and GAG (B). These data are presented as the mean ± SD of three independent experiments. *p < 0.05, **p < 0.01, ***p < 0.001. [file 12951_2020_708_MOESM1_ESM.tif]
